# Letter to the editor concerning the article titled “Mental health status of students during coronavirus pandemic outbreak: A cross-sectional study”

**DOI:** 10.1016/j.amsu.2022.103948

**Published:** 2022-06-06

**Authors:** Fahad Gul

**Affiliations:** Rawalpindi Medical University, Rawalpindi, Pakistan

Dear Editor,

I enthusiastically read an article by Seyyed Muhammad Mahdi Mahdavinoor et al. titled “Mental health status of students during coronavirus pandemic outbreak: A cross-sectional study” published in Annals of medicine and surgery volume 78, June 2022, 103739. Indeed this covid-19 pandemic has increased mental stress, especially among medical students considering their workload. so, I would like to appreciate the author's contribution to measuring the prevalence of anxiety, stress, and depression in medical students of Mazandaran province during covid-19. But, I would like to draw your attention to the exclusion criteria mentioned in this research “students not studying at Mazandaran University of Medical Sciences and Babol University of Medical Sciences”. It would have been better if the author had also excluded students with already diagnosed anxiety/stress/depression students from the study as these students are not depicting the impact of the pandemic on students' mental health and can necessarily alter the results of the study. Considering, that the study was specifically conducted on medical students of Mazandaran University of Medical Sciences and Babol University of Medical Sciences, a comparison of the mental health of students of these institutes should also have been added in the results section.

This article enlightens us on the importance of the mental health of medical students, considering the suggestions mentioned above will make this article more scintillating to read.

## Sources of funding

None.

## Ethical approval

Not required.

## Consent

Not required.

## Author contribution

Fahad Gul: Critical analysis of the article, letter writing.

## Registration of research studies

1. Name of the registry: Not applicable
2. Unique Identifying number or registration ID: Not applicable
3. Hyperlink to your specific registration (must be publicly accessible and will be checked): Not applicable

## Guarantor

Fahad Gul.

## Declaration of competing interest

No conflict of interest to be declared.

